# Gangrenous ischemic colitis localized to the cecum: a case report

**DOI:** 10.1186/s40792-023-01587-x

**Published:** 2023-01-23

**Authors:** Kohei Tateno, Yoko Motegi, Hiroomi Ogawa, Kunihiko Suga, Takuhisa Okada, Katsuya Osone, Ryuji Katoh, Yasunari Ubukata, Hideyuki Saito, Keigo Hara, Makoto Sakai, Kyoichi Ogata, Makoto Sohda, Chiaki Murakami, Ken Shirabe, Hiroshi Saeki

**Affiliations:** 1 Division of Gastroenterological Surgery, Department of General Surgical Science, Graduate School of Medicine, Gunma University, 3-39-22, Showa-machi, Maebashi, Gunma 371-8511 Japan; 2 Department of Human Pathology, Graduate School of Medicine, Gunma University, 3-39-22, Showa-machi, Maebashi, Gunma 371-8511 Japan; 3 Department of General Surgical Science, Graduate School of Medicine, Gunma University, 3-39-22, Showa-machi, Maebashi, Gunma 371-8511 Japan

**Keywords:** Cecal necrosis, Hemodialysis, Ischemic colitis

## Abstract

**Background:**

Ischemic colitis affects the left colon in elderly individuals and localization on the right side, especially in the cecum, is rare. We report a case of gangrenous ischemic colitis localized in the cecum of a patient undergoing hemodialysis.

**Case presentation:**

A 73-year-old man had been undergoing hemodialysis for chronic renal failure caused by diabetic nephropathy. He experienced frequent vomiting, diarrhea, and abdominal pain. Contrast-enhanced computed tomography revealed thickening of the cecal wall, poor enhancement, dilation of the cecum, and intrahepatic portal emphysema. No obvious abnormal findings were observed in the appendix. The patient was diagnosed with cecal necrosis and ileocecal resection was performed. Histopathological examination revealed gangrenous ischemic colitis of the cecum. He was discharged 12 days after surgery without postoperative complications.

**Conclusion:**

It is important to consider the possibility of ischemic colitis of the right colon in the event of renal failure requiring dialysis, to ensure that opportunities for surgical intervention are not missed.

## Background

Ischemic colitis occurs predominantly in the left colon and rarely in the right colon. The manifestations of right-sided ischemic colitis include lower right abdominal pain, vomiting, and loss of appetite. These symptoms closely resemble those of appendicitis, encumbering accurate diagnosis.

The symptomatic presentation and disease course of ischemic colitis of the right colon differ from those of ischemic colitis of the left colon, and the former often requires surgery [1, 2]. Herein, we report a case of gangrenous ischemic colitis located in the cecum in a patient undergoing hemodialysis.

## Case presentation

A 73-year-old man with chronic renal failure caused by diabetic nephropathy presented with acute abdominal pain and frequent vomiting. He had hypertension and dyslipidemia. His medical history included two strokes. Physical examination indicated pain in the lower right quadrant on palpation and rebound tenderness. The laboratory findings were as follows: white blood cell count, 5600/µL; hemoglobin, 13.6 g/dL; platelet count, 138 × 10^3^/µL; serum creatinine, 8.76 mg/dL; creatine phosphokinase, 150 U/L; lactate dehydrogenase, 222 IU/L; and C-reactive protein, 0.24 mg/dL.

**Fig. 1:**
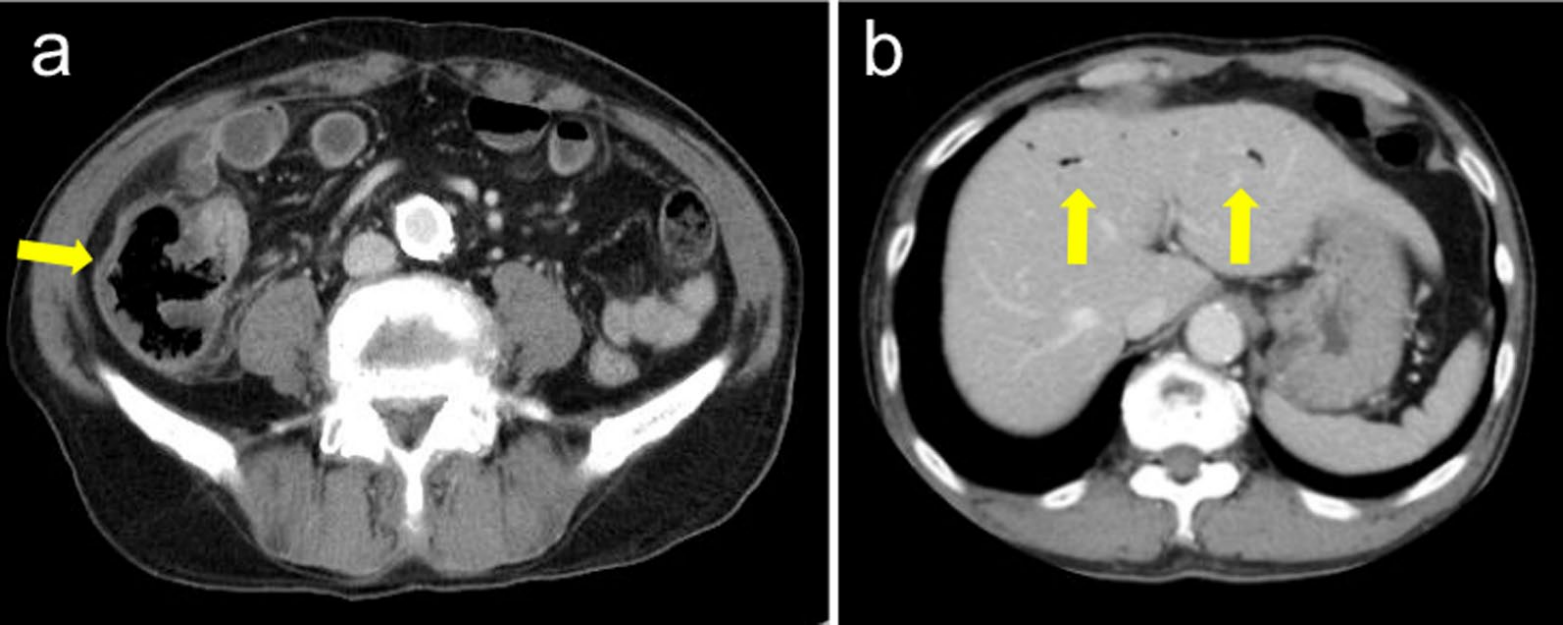
Contrast-enhanced computed tomography. **a** Enhancement of the cecal wall is attenuated (arrow) and an increase in the surrounding fat tissue density is detected. **b** Intra-hepatic portal venous gas is observed (arrow)

Contrast-enhanced abdominal computed tomography (CT) revealed thickening of the cecal wall with poor enhancement, cecal dilation, and intrahepatic portal emphysema. No abnormal findings were observed in the appendix or terminal ileum (Fig. 1).

**Fig. 2:**
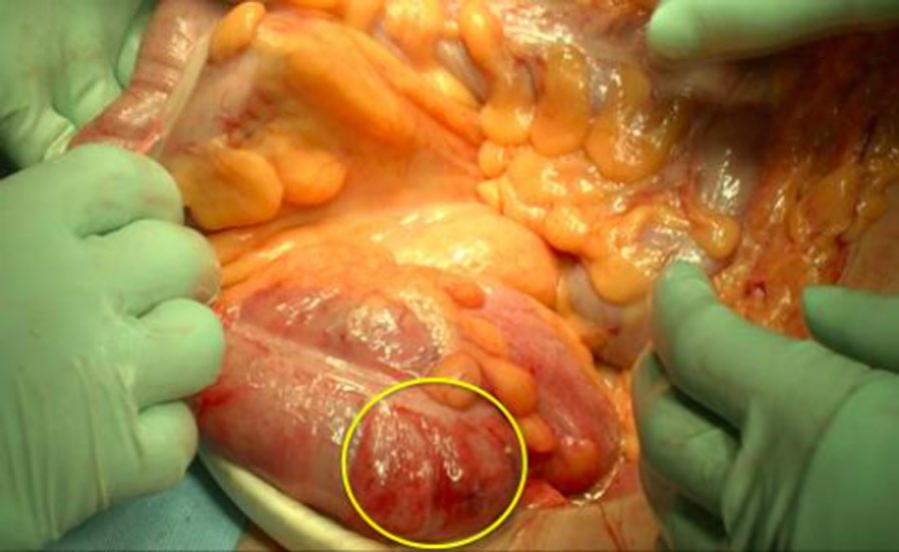
Intraoperative findings. The cecum shows partial discoloration (circle)

**Fig. 3:**
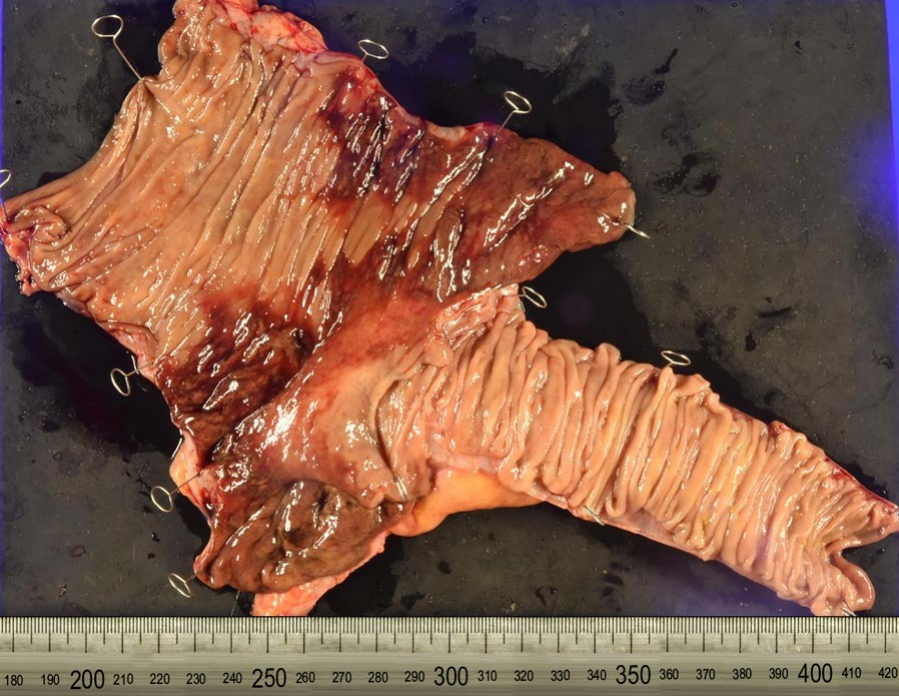
Necrosis and bleeding of the mucosa are observed predominantly in the contralateral mesentery in the ileocecal area

**Fig. 4:**
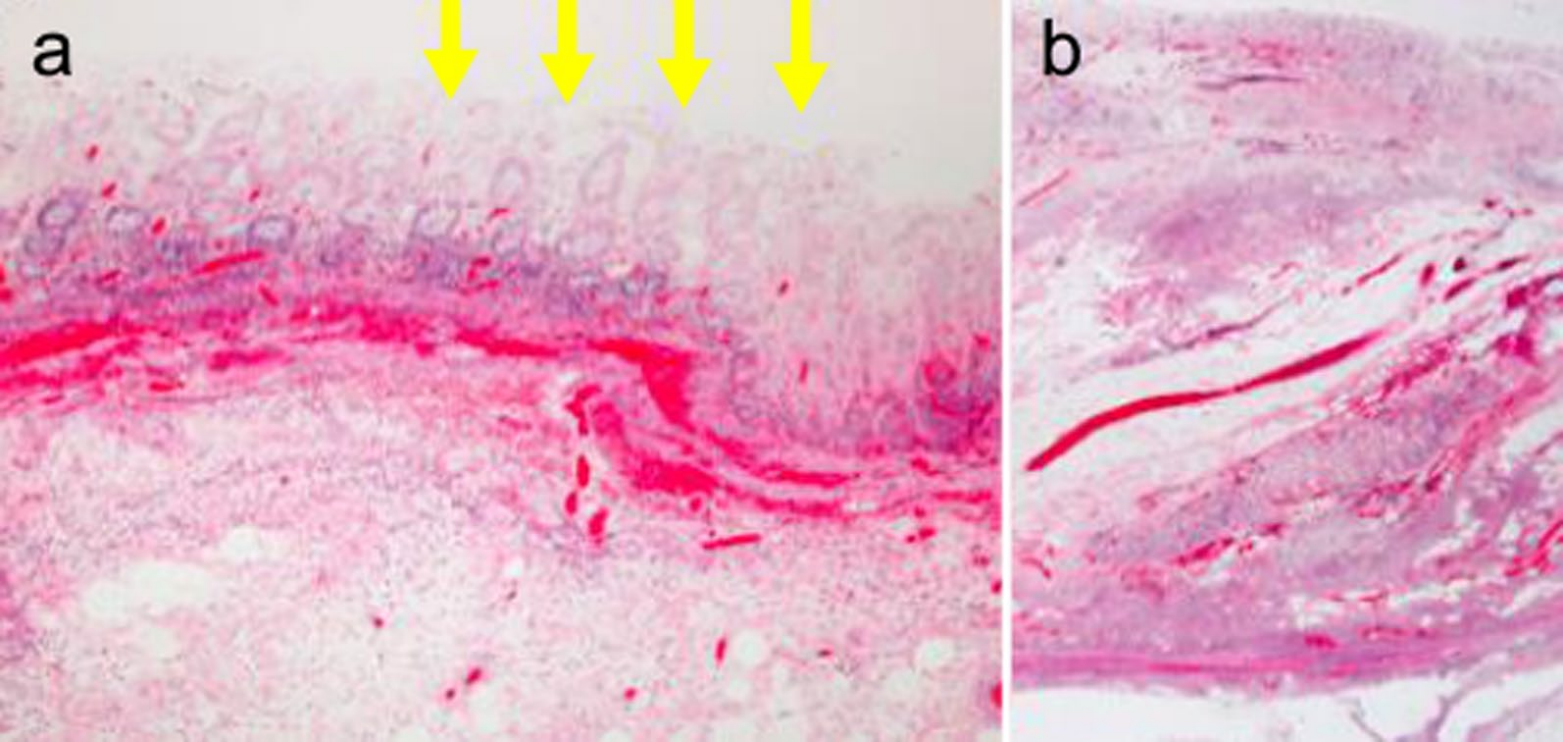
Histopathological examination. **a** A withering necrotic gland duct characteristic of ischemic enteritis is observed (arrow). **b** In the inflamed areas, inflammatory cell infiltration with neutrophils is observed. There is no stenosis of blood vessels or thrombus that could cause intestinal necrosis

We performed emergency laparotomy based on suspicion of cecal necrosis. Partial discoloration of the cecum was observed; therefore, we performed ileocecal resection (Figs. 2 and 3). Pathological examination indicated a necrotic gland duct, which is characteristic of ischemic colitis (Fig. 4). Full-thickness inflammatory cell infiltration consisting predominantly of neutrophils was observed at the site with the highest degree of inflammation, indicating acute inflammation. No vascular stenosis or thrombus causing intestinal necrosis was observed. We diagnosed ischemic colitis with hemorrhagic necrosis of the cecum based on these findings. The patient was discharged 12 days after surgery without postoperative complications.

## Discussion

Ischemic colitis is ischemia of the intestinal mucosa despite the absence of occlusion of the main arteries. This condition often occurs in elderly individuals [3, 4]. The causes of ischemic colitis can be classified as occlusive and non-occlusive [5]. Occlusive factors include atherosclerosis, thromboembolization, venous occlusion, and mechanical bowel obstruction. Non-occlusive cecal necrosis reportedly occurs in association with open-heart surgery, chronic heart disease, certain drugs, and hemodialysis [6–9].

The rectal sigmoid junction and splenic flexure are common sites of ischemic colitis. These sites are frequently associated with “watershed” areas of blood flow. The rectal sigmoid colon forms the boundary between the blood flow from the inferior mesenteric artery and internal iliac artery. Similarly, the splenic flexure constitutes part of the boundary of the superior and inferior mesenteric arteries. Therefore, these areas are anatomically vulnerable to blood supply instability compared to other parts of the colon. In addition to these two areas, the cecum is anatomically prone to ischemia, although this is not well known [10].

The cecum receives blood flow from the cecal arteries, which often branch from the arcade of the colonic and ileal branches of the ileocolic artery [11]. The diameter of the cecum is large; therefore, the lateral wall is at risk for ischemia owing to the long distance from the origin of the cecal artery [12]. Additionally, in some cases, the origin of the cecal artery is more proximal, thus making the cecal wall more prone to ischemia [13].

CT may reveal thickening of the cecal wall, intramural bleeding, focal or diffused increase in the intestinal diameter, mesenteric arterial thrombus, intestinal pneumatosis, portal or mesenteric venous gas, pneumoperitoneum, and intra-abdominal free fluid in patients with cecal necrosis [14, 15]. In our patient, abdominal CT showed thickening of the cecal wall with poor enhancement, cecal dilation, and intrahepatic portal emphysema. Guitart et al. reported that the combination of cecal wall thickening and edematous characteristics accompanied by the lack of changes in the appendix, ileum, and colon is suggestive of a diagnosis of cecal ischemia [16].

In clinical practice, the symptoms and the course of treatment of ischemic colitis vary according to the site of occurrence. In ischemic colitis of the left colon, constipation often triggers ischemia, resulting in bloody diarrhea. Ischemic colitis in the right colon rarely presents with bloody diarrhea; however, vomiting, diarrhea, and right abdominal pain are typical manifestations. Conservative treatment is usually effective for left-sided ischemic colitis. However, surgical treatment is often required for right-sided ischemic colitis because it is more likely to be accompanied by gangrene. The duration of hospitalization was longer (median length of stay, 10 days vs 6 days), and the frequency of surgery (44.3% vs 11.5%) and mortality rate were higher in patients with right-sided colonic involvement compared to those with left-sided colonic involvement [1]. Montoro et al. also reported that mortality and/or the need for surgery were higher for isolated right-sided ischemic colitis compared to ischemic colitis occurring at other sites (40.9% vs 10.3%) [2]. Therefore, right-sided ischemic colitis requires more careful treatment, including surgical intervention.

Our patient had a history of hemodialysis. Studies have reported a relationship between chronic renal failure or hemodialysis and ischemic colitis [3, 14, 17, 18]. Lawrence et al. evaluated 313 cases of ischemic colitis and reported that the frequency of comorbidities, such as coronary artery disease and end-stage renal failure requiring hemodialysis, was higher in right-sided involvement than in left-side involvement [1]. Flobert et al. reported that chronic renal failure, hemodialysis, and right-sided colon involvement are associated with the severity of ischemic colitis [14] and that hemodialysis patients tend to develop ischemic colitis on the right side. Therefore, it is worth noting that hemodialysis patients could develop right-side ischemic colitis and may be prone to serious disease.

Nielsen et al. reported the usefulness of an initial laparoscopic approach in the event of abdominal emergency [19]. In our case, we chose open surgery at the outset, since the patient was undergoing dialysis, and there was a risk of hemodynamic failure due to the long operative duration. However, depending on the patient and circumstances, the laparoscopic approach may be selected initially.

This patient had multiple arteriosclerosis comorbidities, such as hypertension, dyslipidemia, and diabetic nephropathy. We postulated that the hemodynamic changes attributable to hemodialysis were responsible for gangrenous ischemic colitis of the cecum, which is anatomically prone to ischemia.

## Conclusion

It is important to consider the possibility of ischemic colitis of the right colon in cases of renal failure requiring dialysis, to ensure that opportunities for surgical intervention are not missed.

## Data Availability

Data sharing is not applicable for this article because no datasets were generated or analyzed during the current study.

## Abbreviation

CT: Computed tomography
